# The ecology of program director leadership: power relationships and characteristics of effective program directors

**DOI:** 10.1186/s12909-019-1869-3

**Published:** 2019-11-21

**Authors:** Bharat Kumar, Melissa L. Swee, Manish Suneja

**Affiliations:** Division of Immunology, Department of Internal Medicine, University of Iowa, Iowa City, Iowa USA

**Keywords:** Leadership, Medical education, Residency

## Abstract

**Background:**

Program directors are often perceived as strong and independent leaders within the academic medical environment. However, they are not as omnipotent as they initially appear. Indeed, PDs are beholden to a variety of different agents, including trainees (current residents, residency applicants, residency alumni), internal influencers (departmental faculty, hospital administration, institutional graduate medical education), and external influencers (the Accreditation Council for Graduate Medical Education (ACGME), medical education community, and society-at-large). Altogether, these agents form a complex ecosystem whose dynamics and relationships shape the effectiveness of program directors.

**Main body:**

This perspective uses management theory to examine the characteristics of effective PD leadership. We underline the importance of authority, accessibility, adaptability, authenticity, accountability, and autonomy as core features of successful program directors. Additionally, we review how program directors can use the six power bases (legitimacy, referent, informational, expert, reward, and coercive) to achieve positive and constructive change within the complexity of the academic medical ecosystem. Lastly, we describe how local and national institutions can better structure power relationships within the ecosystem so that PD leadership can be most effective.

**Conclusion:**

Keen leadership skills are required by program directors to face a variety of challenges within their educational environments. Understanding power structures and relationships may aid program directors to exercise leadership judiciously towards fulfilling the educational missions of their departments.

## Background

**Fig. 1:**
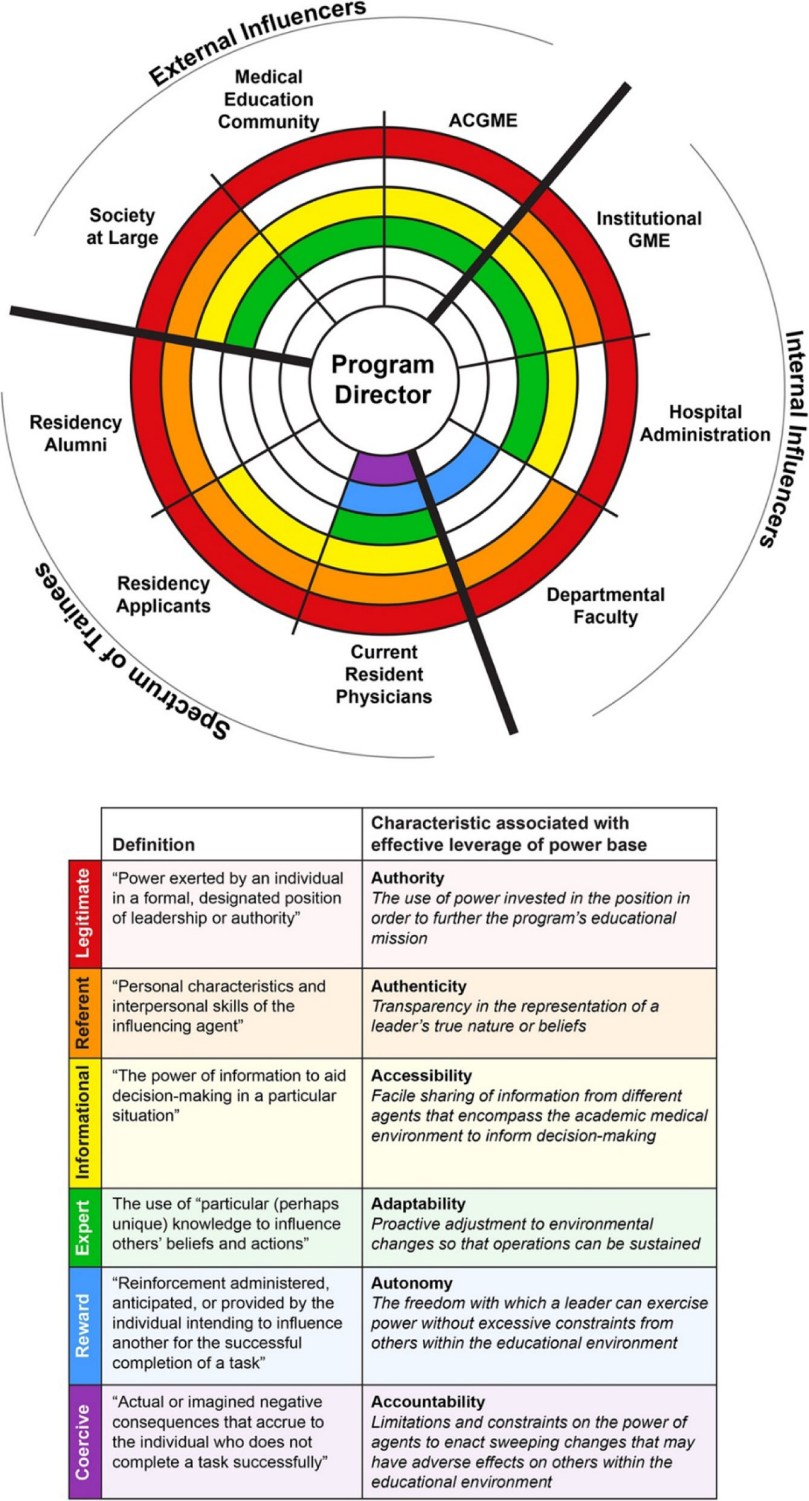
Bases of Power & Characteristics Associated with Effective Program Director Leadership ^5.^ Effective program directors leverage the six power bases to influence other agents within the ecosystem of academic medicine. Legitimate, referent, and informational power bases are the most versatile while reward, coercive, and expert bases must be leveraged in more specialized situations. Appropriate utilization of these power bases defines the characteristics of effective program director leadership

In the eyes of a trainee, the ideal program director (PD) seems to be an omnipotent leader [1]. She or he organizes and implements curricula, evaluates trainee performance, provides meaningful instruction, serves as an important role model, and advocates for trainee’s interests, among other activities. However, they represent a small fragment of the PD’s unique responsibilities as a leader. She or he inhabits a unique niche within a larger ecosystem that interfaces with a number of agents in the residency program, the department, the healthcare system, and the community-at-large (Fig. 1). This creates a set of complex power relationships that influence the characteristics of effective PD leadership, such as authority, accessibility, adaptability, authenticity, accountability, and autonomy (Table 1) [2].

**Table 1:** Agents within the Ecosystem that Influence Program Director Leadership Program directors interact with multiple agents within the ecosystem. Their bidirectional influences are governed by various structures that guide such interactions. Some are formally articulated while others are largely absent

	PD Responsibilities	Guiding Interactions
Accreditation Council for Graduate Medical Education	Execute minimum program requirements stipulated by the ACGME	ACGME Common Program Requirements
Medical Education Community	Contribute to the medical education community through scholarly work and research	ACGME Common Program Requirements
Society-at-Large	Take responsibility for the proper training of providers in the community	None
Institutional GME	Coordinate with local educational officers to implement local policies	Local institutional policies
Internal Medicine Faculty	Arbitrate conflict, distribute resources, and negotiate power relationships with faculty	None
Hospital Administration	Uphold productivity and contribute to financial viability of hospital	Institutional contracts
Current Resident Physicians	Role model professionalism, engage in teaching activities, and promote well-being of residents	ACGME Common Program Requirements
Residency Applicants	Uphold fairness in the application process	National Residency Match Program (NRMP)
Resident Alumni	Promote and advocate for resident alumni interests	None

Currently, PD leadership is not well-defined outside of the Accreditation Council for Graduate Medical Education’s (ACGME’s) Common Program Requirements. This may change in the near future as the ACGME and the Organization of Program Directors Associations hosted a summit to begin to develop consensus on key aspects of successful program directors [3]. Yet even these competencies will need to be contextualized within a larger ecologic framework based on the principles of effective management.

## Main text

### Towards an ecological approach to PD leadership

One of the central tenets of leadership theory is that leadership is not defined by position, but rather by commensal relationships with their followers, whereby they are expected to rise up to the expectations of followers and others within the enterprise [4]. This means that leaders are not as independent and omnipotent as they are perceived by others, or even by themselves [5]. Rather, they live in an ecosystem where power relationships govern the ability to exercise executive authority [ 6]. To understand the role of the PD as a leader, one has to understand the overlapping and conflicting responsibilities within graduate medical education (GME).

While the conception of a leader as a relatively weak figure bristles against heroic notions of leadership, it aligns well with the sociopsychologic conceptions of power. Indeed, if power is defined as the “social influence in which the feelings and/or behaviors of one party are altered or changed through influence of another party,” [3] then there must be a bidirectional and balanced relationship between a leader and her/his followers, depending on the context and situation [7]. For the sake of conceptual simplicity, literature has commonly categorized power as having six different bases: legitimate, expert, informational, reward, coercive, and referent (Figure 1) [8–10].

### Program director authority is defined by more than just position

First, the PD’s position is important simply by virtue of being a position of leadership. There certainly is a fair deal of overlap between the characteristics of leadership exhibited by physicians in general and the leadership that PD’s display. Yet PDs are held to a higher standard than other physicians because they are able to exert the legitimate power uniquely conferred upon them by the variety of agents within the ecosystem. In particular, it is primarily the departmental leaders, institutional Graduate Medical Education (GME) and the ACGME that invest the position upon a particular individual, with other agents bolstering that investiture. In return, one major role of the PD is to work as a delegate for large administrative organizations to trainees and others.

However, the PD’s authority rests not only on having an invested title, but also on the ability to wield the other five bases of power. Informational and expert power bases are exceptionally important in this regard, since they designate the PD as someone with unique powers, duties, and access to important individuals. Compared to other clinicians, program directors are more likely to have specialized training in administrative workflow, either informally ‘on the job’ or formally through Masters programs in Medical Education (MME), Business Administration (MBA), or Hospital Administration (MHA) that distinguish them from other physicians [11].

In turn, PDs may further the authority of departmental and institutional leaders, such as Deans, Vice Deans, Department Chairs, Designated Institutional Officers (DIOs), Hospital CEOs, and PD’s from other divisions/departments, by providing otherwise unobtainable information and expertise. PD’s are considered ‘the face’ of the program to trainees and provide important, sometimes irreplaceable, links to other members of the ecosystem through the exchange of information and expertise. Therefore, the PD’s informational power also rests on her/his ability to obtain and provide information to these individuals, which is highly variable depending on the ecosystem and personalities involved.

This heightened legitimate power also has important implications on the ability to wield reward and coercive power bases. Physicians wield legitimate power as stewards of the healthcare system [12]; likewise, PD’s wield legitimate power as stewards of the graduate medical education system. Compared to other physicians, PD’s have greater latitude in rewarding and/or coercing other members of the healthcare ecosystem, which may also lead to a higher potential for unintended consequences. Therefore, PD’s must exercise their reward and coercive power bases with greater caution.

Furthermore, there is a multidimensional aspect to PD authority. The PD must utilize referent power to negotiate between competing agents inhabiting the same ecosystem. This requires keen interpersonal and communication skills to come to a mutual accord.

### Accessibility advances the informational and referent power bases

However, this important power associated with the PD’s position has a downside. Due to the legitimacy conferred by administration, PDs are often the personification of the educational missions of departments. This may come at the expense of the PD’s accessibility to trainees, since trainees may start to regard the program director as an agent of larger institutional efforts rather as a representative of their own interests.

First, accessibility is important because it strengthens informational power by permitting access to higher quality of information and diversity of voices. This includes information about problems that trainees are experiencing that the program director would not otherwise know, resources within the institution that can be used to advance the department’s educational mission, power dynamics among other members of the ecosystem that may interact either synergistically or destructively, and opportunities to minimize waste while promoting value in healthcare and educational settings. Secondly, by having the PD’s ear, trainees and other individuals within the ecosystem have a greater stake in the program’s success. After all, having an accessible PD helps to uplift the status of trainees from passive recipients of education to more engaged members of the ecosystem [13]. Thirdly, accessibility is a prerequisite to referent power. Since referent power depends on the ability to influence others, there must be a longitudinal relationship that is marked by trust. That can only occur with maintaining accessibility to one’s thoughts and opinions.

### Adaptability is more important than years of experience

The niche of PD leadership is also challenged by the constant changes in the ecosystem. Generally, PDs do not have a permanent designated set of followers. Rather, most residency programs have complete turnover of trainees within three to 5 years. This is in addition to turnovers among faculty and administrative personnel to whom PDs are responsible. Given this constant state of flux, adaptability is a particularly important characteristic of PD leadership [14]. This adaptability doesn’t just refer to relationships with trainees progressing through their education and with staff tasked with upholding the educational mission, but also to execution of duties, refinement of workflow processes, and receptiveness to new educational philosophies, techniques, and technologies.

Historically, stability of leadership has been championed as a method of ensuring continuity. But more recent literature in management suggests that excess stability may be a liability, and may broadly represent stagnation or inflexibility [15]. This change in perspective is important, since PDs tend to be more senior physicians and have relatively long tenures: indeed, over 30% of Internal Medicine Residency PD’s have been in their positions for over 7 years and an equal percentage is above the age of 55 [16]. While other physicians can utilize the length of such experiences as a way to demonstrate and advance their expert power base in clinical medicine [12], that approach may not be as tenable for PD’s. In a world that is changing at an increasingly rapid rate, adaptability, as ambiguous as the term is, may be more important than years of experience. The ACGME’s recommendations seem to be coming around to this conclusion, with a diminishing focus on time-based criteria.

### Authenticity underpins referent powesr

Despite this need for flexibility, the PD is still expected to be a steadfast icon of longitudinal dependability. After all, agents within the ecosystem rely upon the PD for long periods of time. This is particularly true with trainees, where such a relationship should ideally be life-long. Therefore, effective PD leadership demands prioritization of strong and often informal long-term relationships with others inhabiting the ecosystem. That, in turn, requires that the PD demonstrate authenticity.

Acts of authenticity are difficult to demonstrate and appreciate since it is the culmination of very mundane actions that occur over long periods of time. The establishment of trust is one such task that occurs almost imperceptibly [17]. It requires dedication and long-term effort to ensure that the leader is viewed as reliable [18].

Because the PD is considered a role model, it is wholly anticipated that she or he adheres to the professional standards of being a good doctor [19]. This means that program directors must keep their own clinical skills and knowledge up to date, in addition to fostering an environment that allows learners to develop their own clinical skills and knowledge. It also means engaging in benevolence and using good judgment in taking concrete steps to avoid harm to others, such as patients and learners, within the ecosystem. When harm is deemed inevitable, the leader must mitigate that harm and help to prevent such situations from occurring in the future.

Since authenticity is a subjective concept, it is difficult to evaluate. Institutions are under the assumption that PDs are authentic prior to their delegation as PDs, but rigorous evaluation is either not present or not possible after installation. In management literature, there is a trend towards demanding accountability from leaders, particularly in regards to authenticity. The crises of abuse within large institutions have been pinned on the inability of these institutions to recognize inauthenticity and act upon unprofessional behavior. Among the proposed remedies include greater input of followers who are better able to assess authenticity [20].

### Program director leadership demands accountability from multiple actors

Perhaps the most controversial element of leadership is accountability. As much as program directors are expected to demand authority from followers, they too must be held accountable for their actions. Currently, there are several mechanisms and agencies that constrain the abilities of PDs, including ACGME, NRMP, local institutions, departmental leaders, designated institutional officers, and even the law. In contrast, other agents within the ecologic framework have only limited or indirect methods of exacting accountability.

However, this may skew the power dynamics. For example, the ACGME provides specific guidance regarding requirements and responsibilities of training programs. This contrasts with accountability exerted by current trainees, which is largely indirect and not well-defined. That may lead to an inadvertent skewing of priorities, even though both are integral members of the ecosystem.

Once more, the ecological framework of PD leadership can guide efforts by local institutions. In this case, having more holistic input from followers, including trainees, in making decisions regarding the tenure of PDs may be of use. While this may sound alien, it should be seriously considered at the local level since trainees are important agents of the ecosystem in their own right.

### Autonomy enables effective program director self-leadership

Lastly, leadership is not just a transactional exercise among actors within an ecosystem. Rather, leadership begins with self-leadership, which demands soulful reflection and constant struggle towards identification of one’s own deeper self [21]. To confront this uncertainty and transform it into a positive force for change, sufficient autonomy is required so that self-reflection and contemplation can occur without fear of retribution [22].

It must be emphasized that PDs must look after themselves to ensure their own well-being because trainees are uniquely vulnerable to their PD’s well-being. Weak leadership begets weak followership, which, in turn, robs opportunities to observe effective leadership and personal growth [23, 24].

Therefore, local institutions must provide adequate resources and support to guide the PD’s self-leadership. Protection of time and effort represents one such solution that is enumerated by the ACGME. However, it should be considered the bare minimum. Providing coaching from more experienced clinician educators and leaders, funds for formal and advanced training in medical education, hospital administration, or business management, and access to mentorship, counseling, and wellness programs hold the potential to improve the ability of PD’s to leverage power. In this regard, the needs of PD leadership strongly parallel physician leadership [12]. Local institutions should continue to periodically look at the efficacy of PDs in negotiating relationships with other members of the ecosystem and be responsive to PD needs.

## Conclusions

Far from the heroic notions of leadership that are buried within our unconscious minds, the PD actually lives in a unique ecological niche that demands versatility in managing opportunities and crises. Understanding the ecological framework in a way that is based in management theory is vital to ensuring that PDs can uphold the educational missions of their institutions. Moreover, embrace of this framework enables PDs to recognize and utilize power bases to maximum effect in ways that differ from how other physicians use them. This has wide-ranging ramifications, since the well-being of PDs directly impacts the well-being of trainees, who are the future leaders within our profession.

## Data Availability

Not applicable.

## Abbreviations

ACGME: Accreditation Council for Graduate Medical Education
GME: Graduate Medical Education
NRMP: National Residency Match Program
PD: Program Director
